# Preliminary Evaluation of an Injectable Therapeutic for Cisplatin Ototoxicity Using Neuronal SH-SY5Y Cells

**DOI:** 10.3390/medicines12040030

**Published:** 2025-12-09

**Authors:** Michelle Hong, Katherine Kedeshian, Larry Hoffman, Ashley Kita

**Affiliations:** Los Angeles Department of Head and Neck Surgery, David Geffen School of Medicine, University of California, Los Angeles, CA 90095, USA

**Keywords:** cisplatin, ototoxicity, peripheral neuropathy, local drug delivery, polymer microparticle

## Abstract

Background/Objectives: Though ototoxic, cisplatin is a mainstay of chemotherapy for a variety of cancers. One suggested mechanism of cisplatin ototoxicity involves damage to the spiral ganglion afferent neurons in the inner ear. There is a need for a high-throughput model to screen medications for efficacy against cisplatin and to develop a local therapeutic to mitigate cisplatin’s debilitating side effects. Microparticles encapsulating a therapeutic medication are an injectable and tunable method of sustained drug delivery, and thus a promising treatment. Methods: SH-SY5y human neuroblastoma cells were used as a cell line model for the spiral ganglion neurons. The cells were dosed with cisplatin and four potential therapeutics (melatonin, metformin, cyclosporine, and N-acetylcysteine), with cell viability measured by CCK-8 assay. The most promising therapeutic, N-acetylcysteine (NAC), was then encapsulated into multiple poly(lactic-co-glycolic acid) (PLGA) microparticle subtypes of varied lactide–glycolide (L:G) ratios and NAC amounts. The elution profile of each microparticle subtype was determined over two months. Results: Of the therapeutics screened, only cells dosed with 1 or 10 mM NAC prior to cisplatin injury demonstrated an improvement in cell viability (73.8%, *p* < 1 × 10^−8^) when compared to cells dosed with cisplatin alone. The 75:25 L:G microparticles demonstrated an increase in the amount of NAC released compared to the 50:50 L:G microparticles. Conclusions: NAC is a potential therapeutic agent for cisplatin toxicity when tested in a neuronal cell line model. NAC was encapsulated into PLGA microparticles and eluted detectable concentrations of NAC for 6 days, which is a first step towards otoprotection for the weeks long duration of chemotherapy treatment. This work describes a method of screening potential therapeutics and a strategy to develop local drug eluting treatments to protect against cisplatin ototoxicity.

## 1. Introduction

Cisplatin is a widely utilized, highly effective chemotherapy agent used to treat a wide range of malignancies [1,2,3]. Despite its chemotherapeutic efficacy, cisplatin has a debilitating and dose-limiting side effect of ototoxicity [3]. Ototoxicity presents as a progressive, bilateral, and permanent sensorineural hearing loss in at least 60% of patients receiving cisplatin [4]. Even so, cisplatin is still used because it is often the most efficacious chemotherapeutic agent available [1]. The exact mechanism of cisplatin ototoxicity is not fully known [5]. Studies indicate that the primary mechanism involves long-term accumulation of cisplatin in the cochlea, which creates excess reactive oxidative species (ROS) and causes oxidative damage to trigger cell apoptosis [6,7,8]. While hair cell apoptosis is a significant component of cisplatin ototoxicity, cochlear damage is multifaceted and also impacts the afferent spiral ganglion neurons (SGNs) [5,9,10,11,12]. Zheng et al. was among the first to demonstrate that cisplatin has a directly toxic effect on SGNs and that at lower concentrations of cisplatin, there was a greater toxic effect on SGNs than auditory hair cells [11]. The neurotoxic impact of cisplatin on SGNs has been further demonstrated in other animal studies, including mice and guinea pigs [10,11,13]. In human temporal bone histopathology, Hodge et al. found that cisplatin causes a notable decrease in spiral ganglion neurons when compared to control [14]. The afferent neuron is thus a promising therapeutic target within the cochlea. This provides a potential model for screening agents against cisplatin ototoxicity.

The SH-SY5Y cell line is derived from human neuroblastoma and has been well-established as a cell line model for neuronal cells, though not specifically validated as a model of SGNs [15]. Prior studies have utilized SH-SY5Y cells dosed with cisplatin as an in vitro model for cisplatin neurotoxicity and ototoxicity [16,17,18,19]. Donzelli et al. have previously modelled the known peripheral neurotoxicity of cisplatin in SH-SY5Y cell line, demonstrating that cisplatin has dose-dependent toxicity on SH-SY5Y cells [18]. As such, SH-SY5Y cells were utilized in this study to model the damage cisplatin inflicts on afferent SGNs as part of its mechanism of ototoxicity.

There are several important considerations in the search for an effective method of protection against cisplatin ototoxicity. The first of these is identifying a therapeutic agent to utilize. Many different agents have been explored in vivo and in vitro for this purpose. One such agent is N-acetylcysteine (NAC), a reduced glutathione precursor antioxidant that has been effective at reducing cisplatin ototoxicity in in vivo animal and human subject studies at 2% concentration (0.12 M) [20,21,22,23]. Other agents investigated include metformin, an antidiabetic drug shown to prevent oxidative stress-induced cell death; melatonin, a hormone that detoxifies free radicals via electron donation; and cyclosporine, an inhibitor of the Ca^2+^-dependent mitochondrial permeability transition pore [24,25,26]. These have also demonstrated promise in either in vivo or in vitro experiments in auditory cell lines or animal models. These initial studies support screening these potential therapeutic agents for efficacy against cisplatin damage in this SH-SY5Y cell line model.

With many therapeutic agents undergoing research to prevent cisplatin ototoxicity, the method of drug delivery is another important consideration. There is currently great interest in developing a localized and long-term method of drug delivery to the inner ear to mitigate cisplatin ototoxicity without affecting its systemic anti-neoplastic properties [2]. The long-term component of delivery is important, as a typical cisplatin-containing chemotherapy regimen lasts weeks to months and involves cycles of cisplatin infusions often spaced weeks apart. Localized delivery of any drug to the inner ear via transtympanic injection has quite a few difficulties, as any compound injected in the middle ear may drain through the eustachian tube, and multiple intratympanic injections to sustain middle ear concentrations at a level that can diffuse to the inner ear can be uncomfortable for the patient and leave them with a chance of permanent perforation. A potential solution is the utilization of poly(lactic-co-glycolic acid) (PLGA) microparticles as a long-term drug-eluting vehicle to deliver therapeutic agents to the inner ear [27,28]. Many properties of microparticle drug delivery are highly tunable, including elution time, drug encapsulation efficiency, and microparticle size. Thus, this is a highly favorable method of drug delivery for the purposes of mitigating cisplatin ototoxicity.

This study describes a two-fold process in producing a potential therapeutic for protection against cisplatin ototoxicity: (1) the first phase of this study involves screening potentially therapeutic agents with the SH-SY5Y cell line. (2) The second phase focuses on encapsulating one promising agent, NAC, into a PLGA polymer microparticle. This study suggests one process by which future treatments for cisplatin ototoxicity can be developed. This has exciting implications for preserving inner ear function in chemotherapy patients.

## 2. Materials and Methods

### 2.1. Reagents

Product details on reagents and equipment used in this study are listed in Table A1.

### 2.2. Cell Culture

SH-SY5Y human neuroblastoma cells from ATCC were cultured in Dulbecco’s modified Eagle’s medium and F12 medium at a 1:1 ratio supplemented with 10% fetal bovine serum and 1% penicillin-streptomycin. Active cell cultures were incubated at 37 °C and 5% CO_2_. Cells not in active culture were cryopreserved in liquid nitrogen at −200 °C. Cells were checked twice weekly using light microscopy for health and passaged if confluence exceeded 80%. All experiments were performed with cells passaged four or fewer times.

### 2.3. CCK-8 Cell Viability Assay

One method of measuring cell viability utilized was the CCK-8 assay, which uses mitochondrial reduction in a water-soluble tetrazolium salt to an orange formazan crystal. The absorbance from the orange formazan crystal is measured at 460 nm and is directly proportional to the number and metabolic activity of viable cells in the well. SH-SY5Y cells were seeded on a 96-well plate at a density of 3 × 10^5^ cells per well and incubated at 37 °C for 24 h in cell media. When screening a therapeutic for effects on cell viability, the therapeutic was added to the cells at the time of plating and incubated with the cells for 24 h. Well contents were then aspirated, and cells were dosed with 6 µM cisplatin. After 24 h of cytotoxic injury, well contents were aspirated and replaced with fresh media. A total of 10 µL per well of CCK-8 solution was added and incubated at 37 °C for 4 h on an orbital shaker. Finally, absorbance was measured at 460 nm with a microtiter plate reader. Each of the therapeutic agents explored (melatonin, metformin, cyclosporine, and N-acetcylcysteine) was tested at concentrations of at least 5 orders of magnitude based upon solubility and prior studies with each dose replicated in 2 experiments with 6 wells per experiment, totaling 12 replicates per dose. Replicate experiment results were combined by normalizing absorbances to the mean absorbance of the corresponding cisplatin-only control wells. Bioactivity assays were performed by diluting eluted samples of NAC with media to 1 mM prior to dosing.

When testing NAC for its effect on cisplatin injury in SH-SY5Y cells, it was observed that the sulfa group on residual NAC reacted with the CCK-8 solution and potentially confounded the absorbances observed in the initial experiments. This was addressed by adding doses of NAC in media to wells without cells and performing the same CCK-8 protocol described above on the empty wells. The small amount of absorbance detected in these empty wells from the residual NAC was subtracted as “background” from the absorbance of the wells with cells. Each dose of NAC was matched with a corresponding “background” value for that dose. NAC alone was also dosed to identify any effects of NAC alone.

### 2.4. Cell Counting Viability Assay

Another method of measuring viability of cells dosed with NAC and cisplatin was with a cell counting assay. The 2.3 × 10^6^ SH-SY5Y cells were seeded in a 25 cm^2^ cell culture flask and incubated at 37 °C for 24 h in cell media containing 0–10 mM NAC. The media was then aspirated and replaced with media containing 6 µM cisplatin. After 24 h of incubation, the cisplatin-containing media was aspirated and 500 µL Trypsin-EDTA was added to the flask for 1 min. Once cells lifted from the flask surface, trypsin was neutralized with 500 µL cell media. A total of 10 µL of cells was diluted in 10 µL 0.4% Trypan Blue and manually counted with a hemocytometer. Each experiment contained three replicate cell counts per condition.

### 2.5. NAC Microparticle Creation and Size Distribution Analysis

The protocol for creation of microparticles encapsulating NAC was adapted from Murphy et al. [29]. Briefly, 2% PLGA at 50:50 lactide–glycolide (L:G) or 75:25 L:G was dissolved in dichloromethane (DCM). 0, 10, or 20 mg of NAC were then dissolved in 100 µL double-distilled water (ddH_2_O) and added dropwise to the PLGA-DCM solution. The solution was then probe-sonicated on ice at 50% amplitude for 30 s at 5 s pulses. The solution was added dropwise to a solution of 2.5% NAC and 1% polyvinyl alcohol (PVA) and homogenized at 15,000 rpm for 15 min. The homogenized solution was then added to a mixture of 5% NAC and 0.3% PVA and stirred at 700 rpm for 2 h at 40 °C to allow solvent evaporation. The microparticle mixture was then centrifuged at 4500 rpm at 4 °C for 5 min. The supernatants were discarded and the microparticle pellet was resuspended in 2.5% NAC in ddH_2_O. The process of centrifugation and washing in 2.5% NAC in ddH_2_O was repeated twice more. After the final centrifugation, the microparticle pellet was resuspended in ddH_2_O and frozen at −80 °C, then lyophilized overnight and stored at −20 °C.

To evaluate microparticle size distribution, lyophilized microparticles were resuspended in ddH_2_O and placed on a microscope slide. Microparticles were imaged at 63× magnification under transmitted light microscopy (Zeiss, Jena, Germany). Micrographs were analyzed using the *imfindcircles* function in Matlab’s Imaging Processing Toolbox (vR2023a, Mathworks 2023) to automatically detect microparticle diameters. Frequency curves of microparticle diameter distributions were created with R Statistical Software (v1.4.1717, R Core Team 2021).

### 2.6. Microparticle Elution and Encapsulation Efficiency

Microparticle elution was performed in a 12-well Corning HTS Transwell plate with a 0.4 µm-pore membrane. Lyophilized microparticles were resuspended in 500 µL PBS and added to the upper compartment of the transwell apparatus. The microparticles eluted into the collection well below containing 1.5 mL PBS. The apparatus was wrapped in Parafilm to prevent evaporation and placed on an orbital shaker in a 37 °C incubator. Eluted solution from each collection well was removed daily for the first 5 days of elution, then once weekly for the duration of the elution time. This eluted solution was stored at −20 °C.

Encapsulation efficiency of the microparticles is a measure used to estimate the percentage of initial therapeutic agent added during synthesis that became incorporated inside the microparticle. This was calculated by considering the horizontal asymptote of the elution curve the amount of NAC incorporated into the microparticle, and dividing this by the weight of NAC originally added in microparticle synthesis. This calculation assumes that the horizontal asymptote of the elution curve represents complete release of microparticle contents as samples of microparticles imaged using light microscopy at 60 days no longer had appreciable microparticles.

### 2.7. Measuring NAC Concentration of Microparticle Eluent

NAC concentration was quantified with 5,5-dithio-bis-(2-nitrobenzoic acid), also known as Ellman’s reagent, that reacts with the sulfhydryl group on NAC. This produces a yellow color that can be quantified at an absorbance of 412 nm on a microtiter plate reader. This protocol was adapted for use on a 96-well plate and is described in detail in a prior paper [30]. Calibration curves were generated using Ellman’s under the same conditions (in PBS) as the eluted samples. The concentration of eluted samples from empty microparticles synthesized without NAC did not result in concentrations within the limits of NAC detection by Ellman’s reagent.

### 2.8. Statistical Analysis and Figure Creation

Data analyses were performed with R statistical software. Cisplatin dose–response curve was fit to a 4-parameter log-logistic function through the *drc* software package in R. One-way analysis of variance (ANOVA) with Tukey HSD post hoc analysis was used to compare each agent of interest with its corresponding control in the protective agent screening assay. Repeated measures one-way ANOVAs with Tukey’s multiple comparisons post hoc analysis were used to compare cell counts between conditions in the cell counting assay. Two-way ANOVA was used to compare NAC-dosed conditions with and without SH-SY5Y cells. The NAC microparticle elution curve was fit to an exponential curve in R. The assumptions of normality and homogeneity of variance were not assessed.

## 3. Results

### 3.1. Screening for Therapeutic Agents Against Cisplatin Toxicity

**Figure 1:**
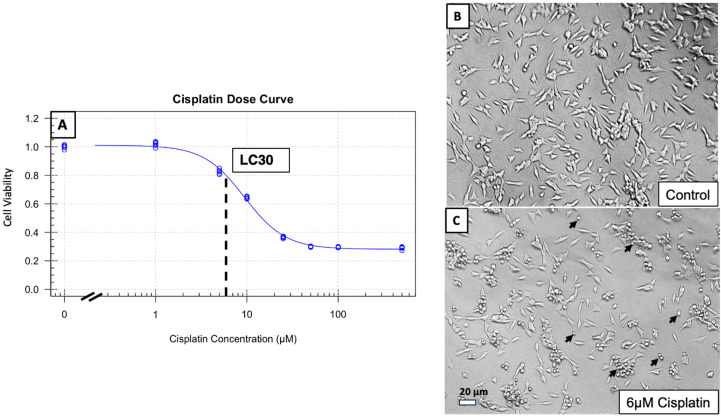
(**A**) Cisplatin dose curve from 0–1000 µM conducted on SH-SY5Y cells. 12 absorbances per cisplatin concentration taken across 2 experiments (6 absorbances per experiment) normalized to each experiment’s average absorbance at 0 µM dose of cisplatin. Dose of cisplatin required to reduce absorbance by 30% (6 µM) indicated by dashed line and LC30 label. 10× light microscopy images of SH-SY5Y cells without cisplatin injury (**B**) and with LC30 dose of 6 µM cisplatin (**C**). Black arrows indicate examples of rounded, non-viable cells in panel (**C**).

The cytotoxic effect of cisplatin on SH-SY5Y cells was quantified with two replicate cisplatin dose curve experiments combined into a normalized curve (Figure 1). Cell viability was evaluated with the CCK-8 assay and cisplatin doses ranged from 0–1000 µM. The dose of cisplatin found to elicit a 30% reduction in cell viability (LC30) was 6 µM, which is within the range of plasma cisplatin levels found in patients undergoing chemotherapy [31].

The LC30 dose of cisplatin provided a reliable, reproducible injury in the SH-SY5Y cell line while allowing for a dynamic range of potential improvement in viability when pre-dosed with therapeutic agents. It was observed that dosing cells at the LC50 of cisplatin often resulted in irreversible injury, thus the LC30 was used for further investigations.

**Figure 2:**
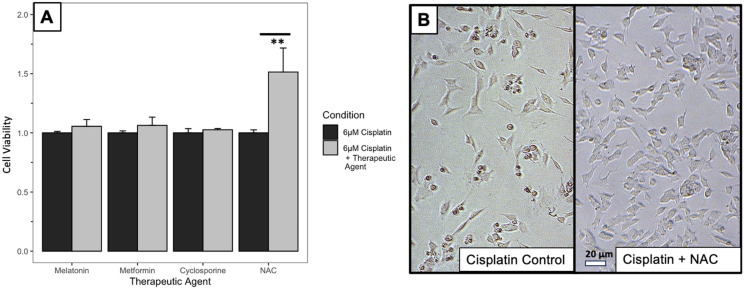
(**A**) Screening therapeutic agents for protection against 6 µM cisplatin injury in SH-SY5Y cells. Cell viability measured via CCK-8 assay, bar values represent the average of 12 normalized absorbances across 2 experiments for a single representative dose of each therapeutic agent (+standard deviation). Black bars represent SH-SY5Y cells injured with 6 µM cisplatin with no therapeutic agent added. Grey bars represent SH-SY5Y cells dosed with a representative dose of a therapeutic agent for 24 h, then injured with 6 µM cisplatin. Representative dose (RD) = dose closest to reaching or dose that reached statistical significance based on *p*-value. Melatonin RD: 1 µM, Metformin RD: 10 µM, Cyclosporine RD: 1 nM, N-Acetylcysteine (NAC) RD: 10 mM. Absorbance is normalized to the mean absorbance of the cisplatin alone condition. ** = *p* < 0.01. (**B**) 10× light microscopy images visually show increased cell density with 10 mM NAC. Cisplatin control = SH-SY5Y cells dosed with 6 µM cisplatin without NAC. Cisplatin + NAC = SH-SY5Y cells dosed with 10 mM NAC for 24 h prior to injury with 6 µM cisplatin.

Next, the potentially therapeutic agents, melatonin, metformin, cyclosporine, and NAC, were added to SH-SY5Y cells 24 h prior to dosing with 6 µM cisplatin, and cell viability was evaluated with the CCK-8 assay. Figure 2A summarizes the results of screening these medications with a representative dose for each therapeutic agent compared to its control condition. Pretreatment with melatonin, metformin, or cyclosporine was not associated with a difference in mean absorbance at any of the doses tested when compared to the control cells dosed with cisplatin alone. Cells pretreated with NAC at 1 mM and 10 mM doses had a 25.8% (*n* = 12, *p* = 1 × 10^−7^) and 73.8% (*n* = 12, *p* < 1 × 10^−8^) increase, respectively, in mean absorbance compared to control cells with cisplatin alone. Micrograph images of the SH-SY5Y cells (Figure 2B) demonstrate visually that cell density is increased when 10 mM NAC was pre-dosed compared to cisplatin injury alone.

### 3.2. Confirmation of NAC Protection Against Cisplatin Toxicity in SH-SY5Y Cells

**Figure 3:**
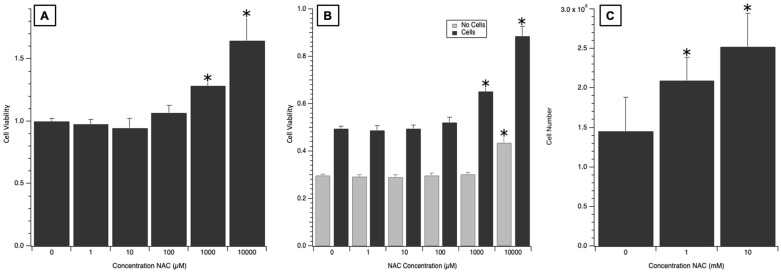
NAC increases cell viability of SH-SY5Y cells in the presence of a cisplatin injury. (**A**) Four replicate experiments of CCK-8 assays resulting in 24 total replicates per NAC dose demonstrate an increase in normalized absorbance with the addition of 1000 µM NAC and 10,000 µM NAC in the setting of 6 µM cisplatin injury. (**B**) While NAC appears to interact with CCK-8 to produce some absorbance at 460 nm, it does not alone account for the full increase in absorbance demonstrated with the addition of 1000 µM and 10,000 µM NAC. (**C**) Cell counting assay corroborates findings of CCK-8 assay. Bar value indicates the average cell count of 3 replicate experiments with 3 counts per experiment (+standard deviation). * = *p* < 0.001.

After NAC was associated with an increase in cell viability in the presence of cisplatin during initial therapeutic screening, the reliability of this result was further confirmed with 4 total replicate CCK-8 experiments with correction for the reaction between the sulfa group and CCK-8 reagent. Aggregated across 4 experiments, cells dosed with NAC demonstrated a 28.3% increase in absorbance at 1 mM NAC *n* = 24, *p* = 1 × 10^−8^) and a 64.6% increase in absorbance in 10 mM NAC *n* = 24, *p* < 1 × 10^−8^) compared to the cisplatin-only control (Figure 3A). An additional experiment was performed with control wells dosed with NAC and cisplatin that did not contain any SH-SY5Y cells to determine whether a reaction between NAC and the CCK-8 compound was confounding the results seen in Figure 3A. This result, illustrated in Figure 3B, showed that while residual NAC did demonstrate a small increase in absorbance at higher doses, it was not enough to fully account for the larger increase in absorbance seen at 1 mM and 10 mM of NAC. Using a cell counting assay, this was confirmed quantitatively with 3 replicate experiments demonstrating a 43.8% (*n* = 9, *p* = 0.005) and 73.2% (*n* = 9, *p* < 0.0001) increase in cell count with 1 mM and 10 mM of NAC added prior to cisplatin injury as compared to no NAC (Figure 3C). 0.1 µM to 10 mM concentrations of NAC alone were also cultured with SH-SY5Y cells and no change in viability was seen compared to control cells not dosed with NAC.

### 3.3. Characterization of NAC-Encapsulated PLGA Microparticle

**Figure 4:**
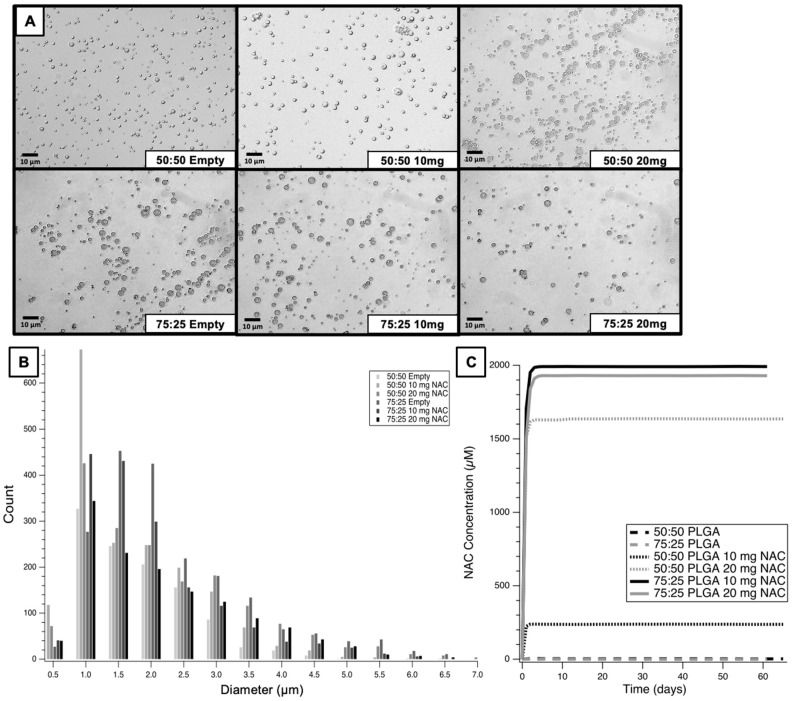
Characterization of NAC encapsulated into PLGA microparticles. (**A**) The 63× light microscope images of PLGA microparticles. “50:50” and “75:25” refer to the ratio of lactide–glycolide of the PLGA used to create the microparticles. Empty = microparticles containing no NAC, 10 mg = microparticles created with 10 mg of NAC added during initial synthesis, 20 mg = microparticles created with 20 mg of NAC added during initial synthesis. (**B**) A custom Matlab script was used to automatically detect over 1000 microparticles per type from multiple microparticle images, which demonstrates the size distribution shown. The majority of microparticles were within 1–2.5 µM. The 50:50 empty microparticles had the smallest diameter and 75:25 empty microparticles had the largest diameter overall. (**C**) Elution curve of NAC microparticles over two months fitted to an exponential function. X-axis break between 10–50 days was performed to direct focus to the earlier elution days and reflects lack of change in eluted NAC beyond day 10.

NAC was thus determined to be a good candidate for PLGA microparticle encapsulation. 50:50 and 75:25 L:G ratios of PLGA polymer were used to synthesize NAC microparticles. There were three subcategories of microparticles (MP) created with each ratio of L:G. The first subcategory was a vehicle control with microparticles synthesized without NAC (empty MP). The second and third subcategories were synthesized with 10 mg (10 mg MP) and 20 mg (20 mg MP) of NAC added at the start of the protocol. There was a total of six types of microparticles created, as seen on light microscopy in Figure 4A. The light microscopy images taken were processed in Matlab, where the diameters of each microparticle were automatically detected and compiled into frequency curves seen in Figure 4B. The most frequent size of each microparticle subtype was in the 1–2 µm range. The largest microparticles created were the 75:25 empty MP and the smallest microparticles were the 50:50 empty MP. The elution curves for each subtype of microparticle are displayed in Figure 4C, with at least 60 days of elution time for all subtypes. The 50:50 and 75:25 empty MPs exhibited no detectable NAC throughout the elution time. The 75:25 10 mg MP had the highest amount of NAC released (1909 µM), as measured by the horizontal asymptote of the fitted exponential curve. The NAC concentrations from the 75:25 10 mg MP and 20 mg MP conditions plateaued at approximately 6 days, while the 50:50 10 mg MP and 20 mg MP conditions plateaued at approximately 2 days. This suggests that the 75:25 PLGA polymer prolongs the release of therapeutic agent compared to the 50:50 PLGA polymer. The encapsulation efficiency (EE) was calculated for every microparticle type. Of the different conditions, the 75:25 10 mg MP had the highest EE of 67.5%. EE of the 50:50 10 mg MP was 7.2%, the 50:50 20 mg MP was 24.2%, and the 75:25 20 mg was 29.6%. After 24 h, the percentage of encapsulated NAC released for each condition was: 87.1% for 50:50 10 mg MP, 87.2% for 50:50 20 mg MP, 87.0% for 75:25 10 mg MP, and 80.1% for 75:25 20 mg MP.

### 3.4. Effect of Eluted NAC on SH-SY5Y Cell Line with Cisplatin Injury

**Figure 5:**
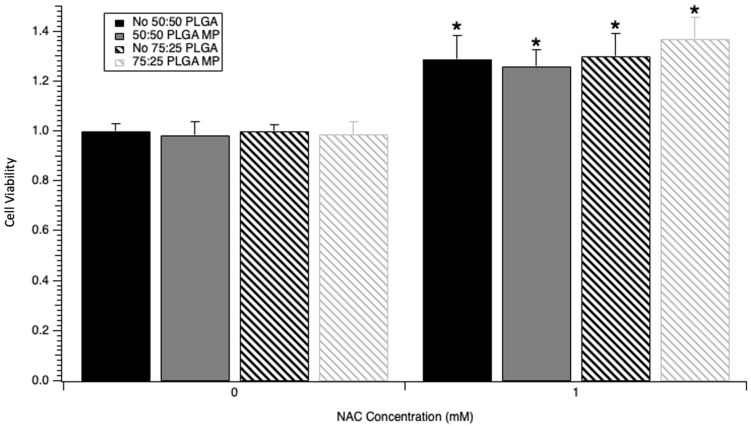
NAC eluted from 50:50 NAC microparticles and 75:25 NAC microparticles has similar effects as freshly prepared NAC on SH-SY5Y cell viability in the presence of a cisplatin injury. Cell viability was assessed with CCK-8 assay, with 12 replicate wells per condition normalized across 2 experiments. All conditions were dosed with 6 µM cisplatin 24 h after plating. (**left**) Cell viability after dosing cells with 0 mM concentrations of NAC (media with PBS for resuspended solutions) or eluted solutions from empty MP not loaded with NAC (50:50 MP Eluent and 75:25 MP Eluent) and (**right**) cell viability after dosing cells with 1 mM concentrations of NAC (resuspended NAC in resuspended solutions) or eluted solutions from MP loaded with NAC (50:50 MP Eluent and 75:25 MP Eluent). * = *p* < 0.001 when compared to respective 0 mM NAC condition.

After creation of the NAC PLGA microparticles, the bioactivity of the NAC eluted from PLGA microparticles was tested and compared to a freshly prepared batch of NAC (stock NAC). SH-SY5Y cells were dosed with NAC eluted from PLGA microparticles and diluted with media to obtain concentrations up to 1 mM prior to cisplatin injury in order to assess retained bioactivity of the eluted NAC with the CCK-8 assay (Figure 5). Since the maximum eluted NAC concentration was 1909 µM, the 10 mM dose of NAC was unable to be assessed despite the efficacy of 10 mM NAC demonstrated in prior experiments. Figure 5 shows a 29.0% increase in CCK-8 absorbance with stock NAC at 1 mM compared to cisplatin-only control (*n* = 12, *p* < 1 × 10^−8^). There was a 28.2% increase in absorbance with 1 mM NAC eluted from the 50:50 microparticle when compared to a vehicle control of 50:50 empty MP eluent dosed at the same volume (*n* = 12, *p* < 1 × 10^−8^). A 37.4% increase in absorbance was seen with 1 mM NAC eluted from the 75:25 MP when compared to its 75:25 empty MP vehicle control (*n* = 12, *p* < 1 × 10^−8^). No difference was seen between conditions of cisplatin-only control and those dosed with the eluent from empty microparticles of each type synthesized without NAC (*p* = 0.897 for 50:50 MP and *p* = 0.962 for 75:25 MP).

## 4. Discussion

### 4.1. Cisplatin Displays Dose-Dependent Toxicity of SH-SY5Y Cells

Cisplatin is known to cause ototoxicity in a dose-dependent manner in patients receiving cisplatin chemotherapy [3]. In this study, the neuroblastoma-derived SH-SY5Y cell line is used as an in vitro model for the inner ear afferent neuron to screen for therapies that may be beneficial against this component of cisplatin ototoxicity. Other studies have previously utilized SH-SY5Y cells to model cisplatin toxicity against neuronal cells, demonstrating a dose-dependent decrease in cell viability when dosed with cisplatin [16,17,18,19]. This study was able to replicate the results of previous literature and demonstrate a reliable dose-dependent injury to SH-SY5Y cells (Figure 1). The dose used to reliably deliver injury to SH-SY5Y cells while still providing a dynamic range for potential recovery from a therapeutic agent was found to be 6 µM. This dose is within the range of cisplatin concentration that is typically seen in the plasma of patients undergoing cisplatin chemotherapy, which can reach 2.76 mg/L (9.17 µM) [31].

### 4.2. NAC Increases SH-SY5Y Viability with Cisplatin Injury

Out of the different agents screened, NAC was the only agent found to be associated with increased SH-SY5Y viability when dosed prior to cisplatin injury. Future studies of the duration of reagent stability and cellular interactions or permeability may explain why metformin, melatonin, and cyclosporine were not found to be protective against cisplatin injury in this SH-SY5Y model. While it has been shown in previous studies that NAC has the potential to reduce cisplatin ototoxicity, these studies showed the effects of NAC in human and animal trials through systemic administration [20,21,22,32]. The utilization of an in vitro model of the afferent neuron allows the unique advantage of high-throughput screening. Cisplatin enacts its deleterious effects via mechanisms that are not yet fully understood, but a primary pathway is thought to be the toxic creation of reactive oxygen species (ROS) and inhibition of antioxidant enzymes, leading to accumulation of ROS and cell apoptosis [7,8,33]. As an antioxidant and a direct scavenger of ROS, NAC is well-suited to protect against this mechanism of cellular damage. NAC was shown by Wang et al. in an in vitro study of ototoxicity to completely protect cisplatin-induced hair cell loss in rat cochlear culture [23]. Results obtained from the SH-SY5Y cell line model supported this literature and thus NAC was encapsulated into a PLGA microparticle.

### 4.3. Encapsulating NAC into PLGA Polymer Microparticles

PLGA microparticles have many advantages as a drug eluting mechanism, as they are highly tunable to the needs of their application [34,35,36]. In this study, NAC was successfully encapsulated into PLGA microparticles as a first step towards the development of a prolonged method of local drug delivery. Several parameters were tested for their impact on the microparticle elution profiles in this study, including the L:G ratio and the amount of NAC loaded into the microparticle mixture. It has been previously demonstrated that higher lactide content can prolong the elution time of the microparticle [29]. This was supported by the results of this study, which showed that the 75:25 10 mg MP and 20 mg MP both had longer periods of NAC elution than the 50:50 counterparts. While the increased elution time of the 75:25 microparticles was insufficient to extend elution time to the length of a chemotherapy regimen, it still showed an improvement that may prove nontrivial when scaling up to human dosing. All the microparticles created underwent significant release within the first 24 h of microparticle elution, which likely limited the amount of NAC remaining for elution. This form of release is well-documented in prior studies of PLGA particles [37,38]. While this may prove to be advantageous in the setting of an acute injury, it may limit the long-term benefits of the loaded therapeutic agent if too much is released at the start of elution.

It was also hypothesized that increasing the amount of NAC initially loaded into the microparticles may assist with prolonging the elution time. There was an increase in amount of NAC released with the 50:50 20 mg MP when compared to the 50:50 10 mg MP, but both formulations had a plateau in release within 2 days of elution. Additionally, the 75:25 10 mg MP and 75:25 20 mg MP both released approximately the same amount of NAC despite more NAC being loaded into the 20 mg MP, and both had a plateau in release around 6 days of elution. The observation that a greater amount of NAC loaded into the 75:25 PLGA microparticles did not result in greater NAC elution suggests that microparticles comprising this 75:25 L:G ratio may have a saturation limit for NAC during microparticle fabrication. The short time frame of release until plateau also suggests that future microparticle modifications may be considered to further prolong release.

### 4.4. NAC Eluted from Microparticle Is Bioactive

While it was observed that PLGA microparticles released NAC, the question remained of whether microparticle-eluted NAC had the same impact on SH-SY5Y cells exposed to cisplatin as freshly prepared NAC. Bioactivity experiments found that eluted NAC was still bioactive as SH-SY5Y cells dosed with eluted NAC of 1 mM showed the same increase in viability as freshly prepared 1 mM NAC in the setting of a cisplatin insult. This result was replicated in both the 50:50 and 75:25 batches of PLGA microparticles. It was also determined that the PLGA vehicle itself did not have any deleterious or beneficial impact on the SH-SY5Y cells when compared to cells dosed with cisplatin alone. This establishes the PLGA microparticle as a viable method of drug delivery that does not compromise the efficacy of the encapsulated agent.

NAC microparticles are one possible therapeutic for the mitigation of cisplatin ototoxicity, as they allow for targeted, prolonged delivery of NAC to the inner ear without interfering with cisplatin systemically. Other studies have observed a positive effect of NAC alone injected intra-tympanically to the round window prior to cisplatin administration [20]. Encapsulation of NAC into a microparticle prior to intra-tympanic injection likely extends the length of drug delivery. SH-SY5Y cells have also been used to model cisplatin-induced peripheral neuropathy [18]. Thus, NAC may also be considered as a potential therapeutic for peripheral neuropathy caused by cisplatin chemotherapy because of the neuronal cell line utilized. While a systematic review conducted by Albers et al. concluded that the current data on NAC is insufficient to determine whether it limits the neurotoxicity of cisplatin, this was based on a systemic method of NAC administration [39]. There may be more therapeutic yield if NAC is delivered locally through a polymer microparticle. More research must be done to further fine-tune this method of drug delivery to develop localized treatments for cisplatin ototoxicity.

Future studies include modeling NAC diffusion and retention in the middle and inner ear in vitro and in vivo to address some of the translational challenges associated with screening therapeutics in individual cell lines, as described here. Additional studies may also include a more detailed mechanistic evaluation of NAC’s protective effect and modification of microparticles to further prolong release.

## 5. Conclusions

The SH-SY5Y neuronal cell line was used to model the ototoxicity that results from cisplatin injury to the afferent neurons of the inner ear. Cisplatin was found to have a dose-dependent cytotoxic effect on SH-SY5Y cells. SH-SY5Y cells dosed with cisplatin were used as a screening system to successfully identify a therapeutic agent of interest, NAC, that protects SH-SY5Y cells against cisplatin cytotoxicity. NAC was then successfully encapsulated into PLGA polymer microparticles. NAC eluted from microparticles was shown to have the same protective effects as freshly resuspended NAC. This work describes one method of screening therapeutics and developing targeted, long-term drug eluting treatments to protect against cisplatin ototoxicity.

## Data Availability

Data regarding this study can be provided upon request.

## Abbreviations

The following abbreviations are used in this manuscript:

NAC	N-acetylcysteine
PLGA	Poly(lactic-co-glycolic acid)
MP	Microparticles

## Appendix A

**Table A1:** Product information for all materials and reagents used in this study.

Reagent/Product	Vendor	Vendor Location	Product Number
Cisplatin	Cayman Chemical	Ann Arbor, MI, USA	13119
N-acetylcysteine	Sigma-Aldrich	Saint Louis, MO, USA	A7250
Melatonin	Alfa Aesar	Ward Hill, MA, USA	J62452-06
Metformin	Sigma-Aldrich	Saint Louis, MO, USA	PHR1084
Cyclosporine	Biotang Inc.	Lexington, MA, USA	50-751-3695
SH-SY5Y cell line	ATCC	Manassas, VA, USA	ATCC-CRL-2266
1:1 Dulbecco’s modified Eagle’s medium and F12 medium	ThermoFisher Scientific	Waltham, MA, USA	11320033
Fetal Bovine Serum	ThermoFisher Scientific	Waltham, MA, USA	26140079
Penicillin-streptomycin	ThermoFisher Scientific	Waltham, MA, USA	15140122
CCK-8	Sigma-Aldrich	Saint Louis, MO, USA	96992
T25 Cell Culture Flask	Falcon	Glendale, AZ, USA	353109
Trypsin-EDTA	ThermoFisher Scientific	Waltham, MA, USA	25200056
Trypan Blue	ThermoFisher Scientific	Waltham, MA, USA	15250061
50:50 lactide–glycolide PLGA	Corbion	Lenexa, KS, USA	PURASORB 5004A
75:25 lactide–glycolide PLGA	Corbion	Lenexa, KS, USA	PURASORB 7504A
Dichloromethane	Sigma-Aldrich	Saint Louis, MO, USA	L090000
Polyvinyl Alcohol	Sigma-Aldrich	Saint Louis, MO, USA	341584
12-well transwell plate	Corning HTS	Durham, NC, USA	3401
Ellman’s Reagent	ThermoFisher Scientific	Waltham, MA, USA	22582
